# Comparison of Pathogenicity of Different Infectious Doses of H3N2 Canine Influenza Virus in Dogs

**DOI:** 10.3389/fvets.2020.580301

**Published:** 2020-11-13

**Authors:** Yongbo Liu, Cheng Fu, Gang Lu, Jie Luo, Shaotang Ye, Jiajun Ou, Xiangbin Wang, Haibin Xu, Ji Huang, Liyan Wu, Xin Zhang, Peixin Wu, Shoujun Li

**Affiliations:** ^1^College of Veterinary Medicine, South China Agricultural University, Guangzhou, China; ^2^Guangdong Provincial Key Laboratory of Prevention and Control for Severe Clinical Animal Diseases, Guangzhou, China; ^3^Guangdong Provincial Pet Engineering Technology Research Center, Guangzhou, China; ^4^Institute of Animal Science and Technology, Zhongkai University of Agriculture and Engineering, Guangzhou, China

**Keywords:** canine influenza virus, H3N2, beagle, minimum infectious dose, pathogenicity

## Abstract

The canine influenza virus (CIV) outbreaks have raised concerns as they pose a threat to the health of dogs. The successful construction of a canine influenza (CI) infection model is essential to study the CIV. Here we investigated the pathogenicity of different infectious doses of H3N2 CIV in Beagle dogs. Thirty-seven healthy Beagle dogs were used in the experiment and were infected with 10^3^, 10^4^, 10^5^, and 10^6^ 50% egg-infectious doses (EID_50_). Compared to the dogs in the other three groups, those in the 10^6^ EID_50_ group presented with obvious clinical symptoms, high virus titer, and typical pathological changes. Considering the ensemble of clinical scores, body temperature, virus shedding, lung lesions, pathological section scores, and visceral virus titers, we determined that 10^6^ EID_50_ is the minimum infectious dose for the Beagle infection model. The other three infectious doses had almost no clinical symptoms. These results indicate that 10^6^ EID_50_ is the minimum infectious dose of H3N2 CIV that can cause obvious clinical manifestations and pathological changes associated with CI in Beagle dogs. The theoretical framework developed in this research will guide the establishment of an infection model of CIV for future research.

## Introduction

Canine influenza virus (CIV) belongs to the family *Orthomyxoviridae* and contains eight single-stranded negative-sense RNA segments that encode more than 15 viral proteins (1). In recent years, it has posed a serious threat to the health of dogs and public health security. The avian-origin H3N2 CIV was first discovered in southern China (2), then spread quickly to other parts of Asia (3), and broke out in the United States in 2015 (4). It is worth noting that, H3N2 CIV can recombine with other influenza viruses (IAVs). It was found that natural co-infection with H3N2 CIV and H1N1 pdm09 virus in a single host could result in a wild-type H3N1 virus (5). In addition, in 2015, a novel H3N2 CIV carrying the polymerase acidic (PA) gene from the H9N2 avian influenza virus was isolated in South Korea (6). Therefore, dogs are potential mixing vessels of influenza viruses (7). Owing to the susceptibility of dogs to infection and their close physical contact with humans, the risks for human infection with new influenza strains increases exponentially (8).

Clinical symptoms caused by influenza virus infections vary widely and depend on the route of inoculation and the immune status of the host (9). Different researchers have used different doses of H3N2 CIV to test Beagle dogs for animal infection models. Hong et al. injected Beagles with 2 × 10^6^ 50% egg-infectious doses (EID_50_) of H3N2 CIV to study the clinical symptoms of influenza virus infections (10). Su et al. injected 10^6^ EID_50_ of H3N2 CIV into Beagle dogs to study proteomics changes triggered by CIV (11). Lyoo et al. inoculated dogs intranasally with 10^6.5^ EID_50_ of H3N2 CIV to build a model for CI infection (12). However, there is no systematic study on the minimum infectious dose of H3N2 CIV that causes severe clinical symptoms in Beagles.

The successful construction of an infection model is essential for the study of CIV. Jirjis et al. (13) and Deshpande et al. (14) established a scoring system for clinical signs to study H3N8 CIV. Luo used this scoring system for clinical signs of respiratory disease when studying H3N2 CIV (15). We found that the clinical sign scores of Beagles with CI symptoms were >3 in all three studies. Bodewes infected Beagles with 10^6^ TCID_50_ of H3N2 CIV and found that all dogs with obvious clinical signs had an affected lung area >10% (9). Hence, we believe that referring to this standard clinical scoring method, a model that initiates an infection with a clinical score >3 and an affected lung area >10% can be considered a successful H3N2 CIV infection model. However, the minimum infectious dose of H3N2 CIV to develop CI has not yet been systematically investigated. In this study, we infected Beagles with different doses of H3N2 CIV and evaluated the body temperature, clinical scores, virus titers, area of the affected lung, and histopathology scores with the aim of finding the minimum challenge dose of H3N2 CIV. This study could provide valuable information for constructing the H3N2 CIV infection model for further studies on CIV.

## Materials and Methods

### Virus

The A/canine/Guangdong/04/2014 (H3N2), referred to as the GD14 strain, was isolated from a pet dog in Guangdong province, China. The virus was propagated in 9-day-old specific-pathogen-free (SPF) embryonated chicken eggs (Wenshi Group Co., Ltd.) at 37°C for 48 h and stored at −80°C. Viral titers were evaluated by EID_50_/mL and calculated by the Reed-Muench method (15).

### Clinical Studies and Virus Challenge

Thirty-seven (10-week-old) Beagle dogs were obtained from the Fuzhou Zhenhe experimental animal Co., Ltd. (China). Prior to the experiment, serum samples were collected from all dogs and tested by hemagglutination inhibition (HI) assays to ensure that the animals had not been exposed to CIV H3N2. Five dogs were used as controls. For intranasal administration, the dogs were divided into 4 groups of 8 and inoculated intranasally with 1 mL of 10^3^, 10^4^, 10^5^, or 10^6^ EID_50_ of GD14. The control group was inoculated intranasally with the same volume of sterile phosphate-buffered saline. The dogs were housed in the negative pressure room of the Animal Experimental Center of South China Agricultural University. Each dog was housed in a separate cage. They were observed for 1 week and fed regularly before starting the experiment.

**Figure 1:**
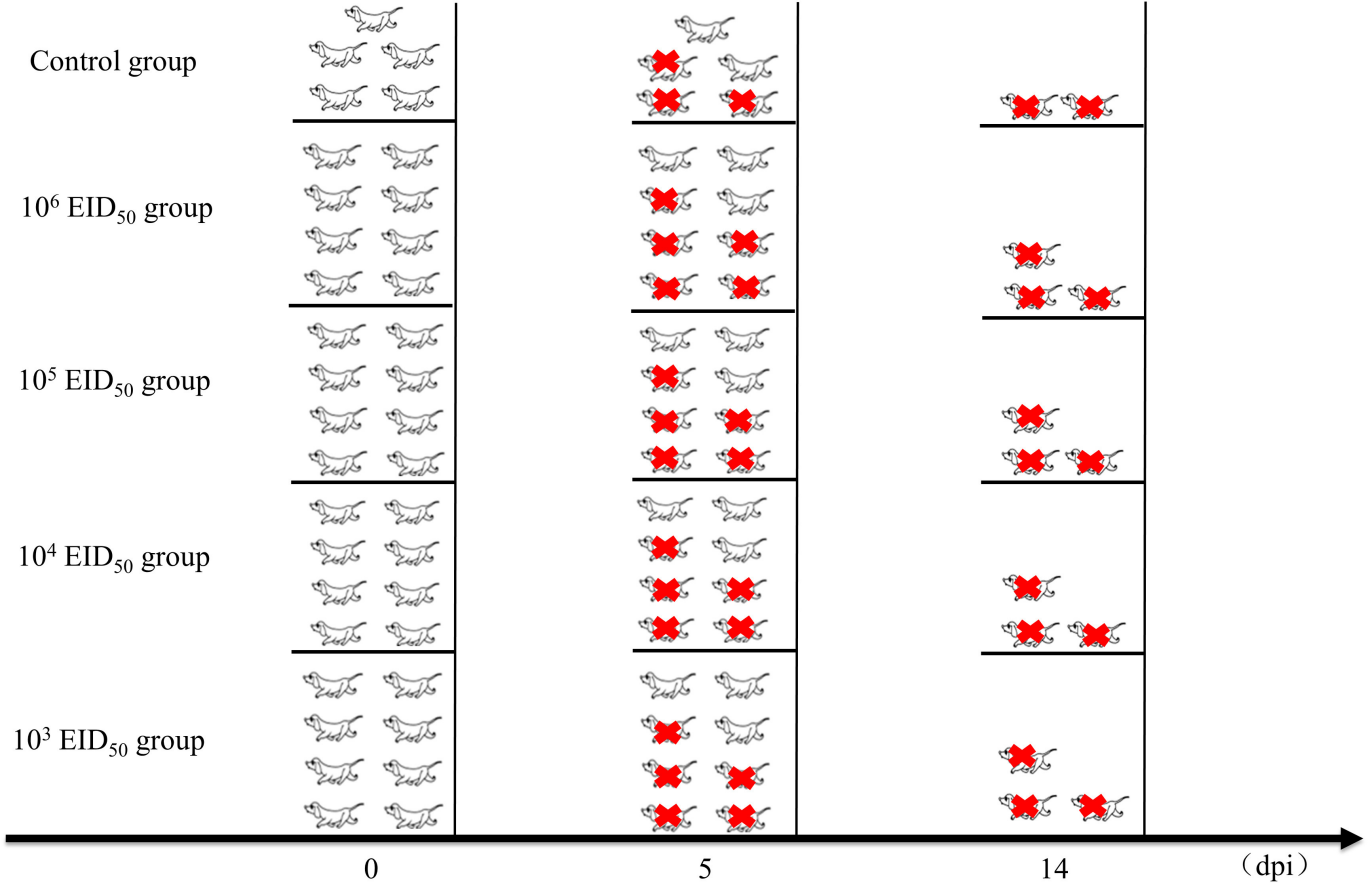
Clinical studies and experimental grouping. Five dogs were used as controls. For intranasal administration, the dogs were divided into 4 groups of 8 and inoculated intranasally with 1 mL of 10^3^, 10^4^, 10^5^, or 10^6^ EID_50_ of A/canine/Guangdong/04/2014. The control group was inoculated intranasally with the same volume of phosphate-buffered saline. Three dogs from the control group and five from each experimental group were euthanized at 5 dpi. After the experiment, all remaining Beagles were euthanized. The red icons refer to euthanasia.

After inoculation, the rectal temperature of the dogs was measured at 10:00 every day. Nasal swabs were also collected, during which the clinical symptoms of the dog were monitored. The clinical score of every dog was evaluated using the previously described scoring system (13) (Table 1). Nasal secretions were collected from the left and right nostrils of each animal every day until 10 days after inoculation (0–10 dpi) and diluted with 1 mL of PBS containing 1% Penicillin and streptomycin. The nasal swabs were used for measuring the EID_50_ using 9–11-day-old embryonated chicken eggs (Wenshi Group Co., Ltd.). Three dogs from the control group and five from each experimental group were euthanized at 5 dpi. Turbinates, tracheas, and lung tissues were collected and the EID_50_ in each sample was determined. Briefly, 1 mL of sterile PBS was added per g of collected tissue, which was then grinded in a liquid nitrogen homogenizer. The supernatant was collected by centrifugation and inoculated in 9-day-old chicken embryos with different dilution concentrations. After incubation at 37°C for 48 h, the hemagglutination titer was determined, and the EID_50_ was calculated by the Reed-Muench method. Animal health and behavior were monitored daily in order to implement euthanasia measures (Humane Endpoint) immediately on animals if the following occurred: complete loss of appetite for up to 5 days or poor appetite (<50% of normal amount) for up to 7 days, showing depression and hypothermia (<37°C) without anesthesia or sedation. After the experiment, all remaining Beagles were euthanized (Figure 1).

**Table 1:** Clinical symptom score criteria.

**Symptom**	**Degree**	**Score**
Coughing	Asymptomatic	0
	Twice (within 10 min)	1
	More than twice (within 10 min)	2
Nasal and eye secretions	Asymptomatic	0
	Mild	1
	Medium	2
	Serious	3
Breathing	Normal	0
	Abnormality	1
Mental condition	Normal	0
	Abnormality	1
Appetite	Normal	0
	Loss of appetite	1
Sneezing	Asymptomatic	0
	≤3 times/10 min	1
	>3 times/10 min	2

Clinical symptoms were observed on days 0–9 dpi and scored according to the following criteria. The final total score was calculated as follows (15):

*(Sneezing score) + 2× (cough score) + 2× (mental condition score) + 2× (appetite score) + 2× (breathing score) + (nasal and eye secretions score)*.

### Serological Testing

Blood samples were collected from the dogs at days 0, 3, 6, 9, 12, and 14 dpi. Approximately 500 μL of serum was collected from each dog and stored at −20°C until use. First, 1 volume of serum was mixed with 3 volumes of receptor destroying enzyme (RDE, Denka Seiken Co., LTD.) and incubated for 18 h at 37°C followed by 30 min at 56°C. The antiserum titer was then determined by the hemagglutination inhibition assay (16). The 1% red blood cells used in this experiment were collected from specific pathogen-free cocks and diluted using sterile PBS.

### Histopathological Examination and IHC Analysis

Lung tissues were fixed in 10% formalin for more than 48 h. Samples were washed overnight, dehydrated with alcohol, and embedded in paraffin. Next, the paraffin-embedded tissues were cut into 4–7-μm-thick sections and deposited onto glass slides. These were then mounted and left overnight at 37°C, prior to hematoxylin and eosin (H&E) staining. Antigen distribution in tissue was detected by immunohistochemistry. Briefly, tissue sections were incubated at 4°C for 12 h with a mouse polyclonal antibody against the H3N2 CIV nucleoprotein (primary antibody; preserved by our laboratory). Then, the sections were incubated with a horseradish peroxidase goat anti-mouse IgG (H + L) antibody (secondary antibody; Abbkine, Wuhan, China) at 25°C for 50 min and finally stained with diaminobenzidine. The development of an amber hue indicated positive staining.

### Gross Pathology and Histopathology Scoring

For pathology analysis of the lungs, the percentage of the affected lung area was assessed by visual inspection (15, 17). A total of 11 and 12 points were assigned to the right and left anterior lobe, respectively, 25 points (13 for the left and 12 for the right) to the cardiac lobes, 10 points to the accessory lobe, and 20 and 22 points to the left and right caudal lobes, respectively, to reach a total of 100 points (Figure 4A). Each HE section was randomly selected from five fields using an optical microscope with a 10× objective lens to score the size and severity of the inflammatory lesions (9). Dimensions of the inflammation area per field that were ≤10×, >10× and ≤2.5×, and >2.5× of the objective lens were scored as 1, 2, 3, respectively. The severity of inflammation was expressed as follows: 1 for “mild,” 2 for “moderate,” and 3 for “obvious.” The average cumulative value of the size and severity of inflammation was used as the histopathological score for each dog.

### Clinical Symptom Score Criteria

Clinical symptoms were observed on days 0–9 post-infection and scored according to the following criteria with the final total score calculated as follows (15):

(Sneezing score) + 2× (cough score) + 2× (mental condition score) + 2× (appetite score) + 2× (breathing score) + (nasal and eye secretions score). The detailed clinical symptom score criteria are shown in Table 1.

### Statistical Analyses

Statistical significance was determined using the conventional Student's *t*-test results. The differences in means were tested using the one-way ANOVA with *post-hoc Tukey's* multiple-comparison test (GraphPad Software, Inc., La Jolla, CA). A *p* < 0.05 was considered significant (^*^p < 0.05; ^**^p < 0.01; ^***^p < 0.001; ^****^p < 0.0001).

### Ethics Approval

All procedures in animal experiments were supervised and inspected by the Experimental Animal Ethics Committee of South China Agricultural University [SYXK (YUE) 2014-0136]. All experimented animals were monitored by university-licensed veterinarians. Ethical approval for this experiment was obtained from the Laboratory Animal Center of South China Agricultural University. Laboratory experiments were conducted under biosafety level 2 conditions, with investigators wearing appropriate personal protective equipment.

## Results

### Clinical Symptoms

**Figure 2:**
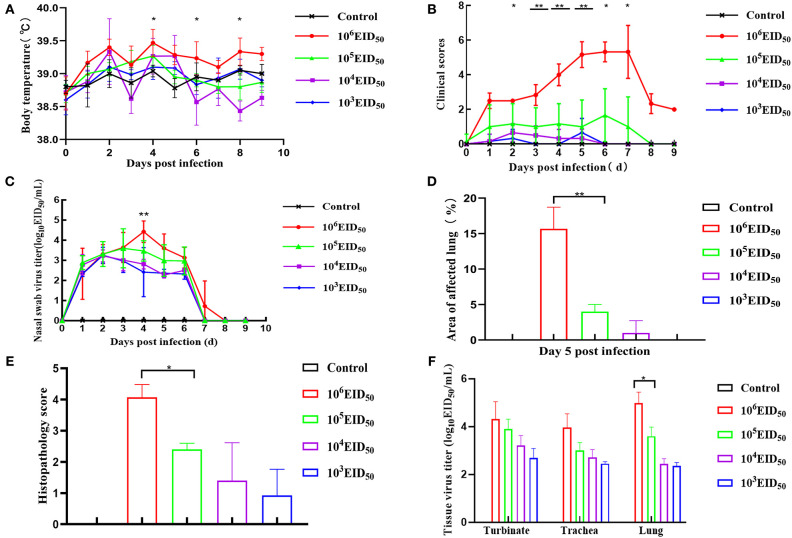
Clinical symptoms. Dogs were inoculated with 10^6^, 10^5^, 10^4^, and 10^3^ EID_50_ (*n* = 8) of GD14 virus or PBS (*n* = 5) as control. Nasal turbinate, tracheal, and lung tissues were collected after euthanizing three dogs in every group at 5 dpi. **(A)** Body temperature was recorded at days 0–10 dpi; **(B)** after infection with H3N2 CIV, days 0–10 clinical scores were recorded based on previous criteria (15); **(C)** nasal swabs were determined by EID_50_ assay. **P < 0.01 indicates that at 4 dpi, the nasal swab virus titer of the 10^6^ EID_50_ group was extremely different from that in the 10^5^ EID_50_ group; **(D)** lung lesion score; **(E)** histopathology score. **P < 0.01 indicates that at 5 dpi, the lung lesion scores of 10^6^ EID_50_ were extremely significantly higher than that of 10^3^ EID_50_. **(F)** Tissue virus titer. An EID_50_ assay was conducted to evaluate the virus titers in the turbinate, tracheal, and lung tissues at 5 dpi (*n* = 5 per group). Error bars represent the standard errors of the mean; *p*-values using Student's *t*-test are indicated.

Compared to the control group, the body temperatures of the dogs in the four challenge groups showed varying degrees of fluctuations, and there was fever at 2 and 4 dpi. When the infectious dose was 10^6^ EID_50_, the dogs' body temperature was higher than the other three challenge groups at all points in time. There was a significant difference when the dose of 10^5^ EID_50_ was compared at 4, 6, and 8 dpi (*p* < 0.05). Nevertheless, the other three groups only showed mild body temperature increases (Figure 2A). After infection with a virus titer of 10^6^ EID_50_, all the dogs showed some respiratory symptoms at 2 dpi, including sneezing, clearing of the nose, as well as coughing on the 3rd day after inoculation; the symptoms continued until day 6. The clinical symptoms in the other three groups were slightly milder. Nevertheless, when the infectious dose was 10^4^ EID_50_ and 10^3^ EID_50_, almost no clinical symptoms were observed. When the infectious dose was 10^6^ EID_50_, the clinical symptoms scores were higher than in the other three groups at 0–8 dpi and were >3 at 4, 5, 6, and 7 dpi. Comparing the infectious dose at 10^6^ EID_50_ with 10^5^ EID_50_, the clinical symptom scores of dogs were extremely different at 3, 4, and 5 dpi (*p* < 0.01). The difference was significant at 2, 6, and 7 dpi (*p* < 0.05) (Figure 2B). None of the animals met the Humane Endpoint during this study.

### Virus Titers

The virus was detected within 48 h post-infection, and virus shedding via rhinorrhea lasted for 6–7 days in all the challenged dogs. On the third day after inoculation, the virus titer reached its highest level. The virus titer of the 10^6^ EID_50_ group was higher than the other three groups at 4 dpi, and the virus shedding in the 10^6^ EID_50_ group was significantly higher than that in the 10^5^ EID_50_ group (*P* < 0.01) (Figure 2C). No virus was detected on the 8th day, showing that virus shedding terminated at 8 dpi in all dogs. As shown in Figure 2F, virus titers were evaluated from turbinates, tracheas, and lungs of infected dogs at 5 dpi. Samples of all three tissues from the 10^6^ EID_50_ group had the highest virus titer among those from all experimental groups. Nevertheless, tracheas and lungs had higher virus titers than turbinates among the three groups. This suggests that the CIV replicated more easily in the trachea and the lungs. The virus titers in the lungs were significantly different between the 10^5^ EID_50_ and 10^6^ EID_50_ groups (*P* < 0.05).

### Seroconversion (HI Titer)

**Figure 3:**
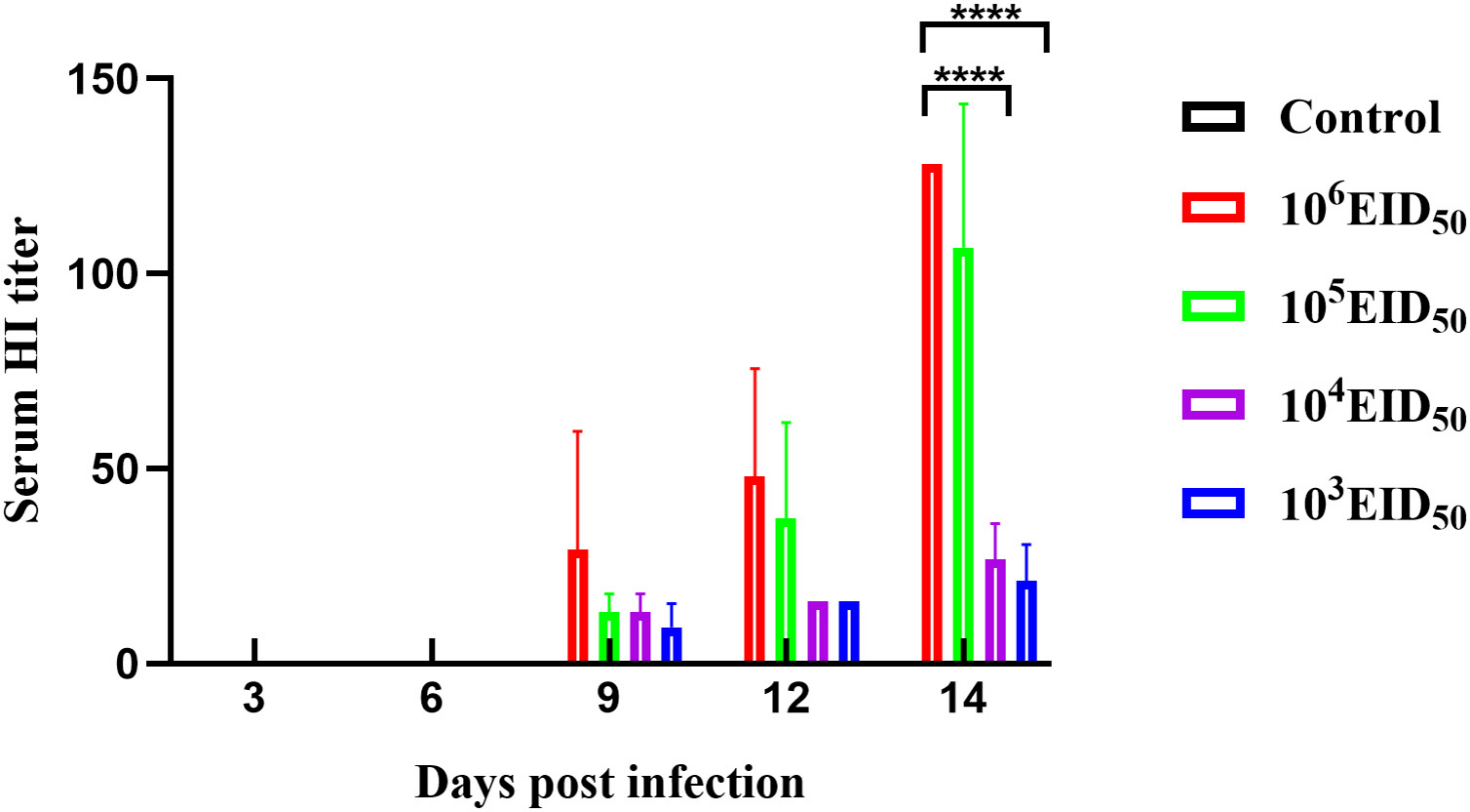
A HI titer was conducted to evaluate the sera specific antibody levels. Error bars represent the standard errors of the mean.

Low antibody levels were detected in canine sera at 9 dpi. At 9, 12, and 14 dpi, the serum antibody levels of the 10^6^ EID_50_ group were higher than those of the other three experimental groups. The antibody levels at 14 dpi in the two groups, 10^3^ EID_50_ and 10^4^ EID_50_, were significantly different from those in the 10^6^ EID_50_ group (*P* < 0.0001) (Figure 3).

### Anatomical Examination

**Figure 4:**
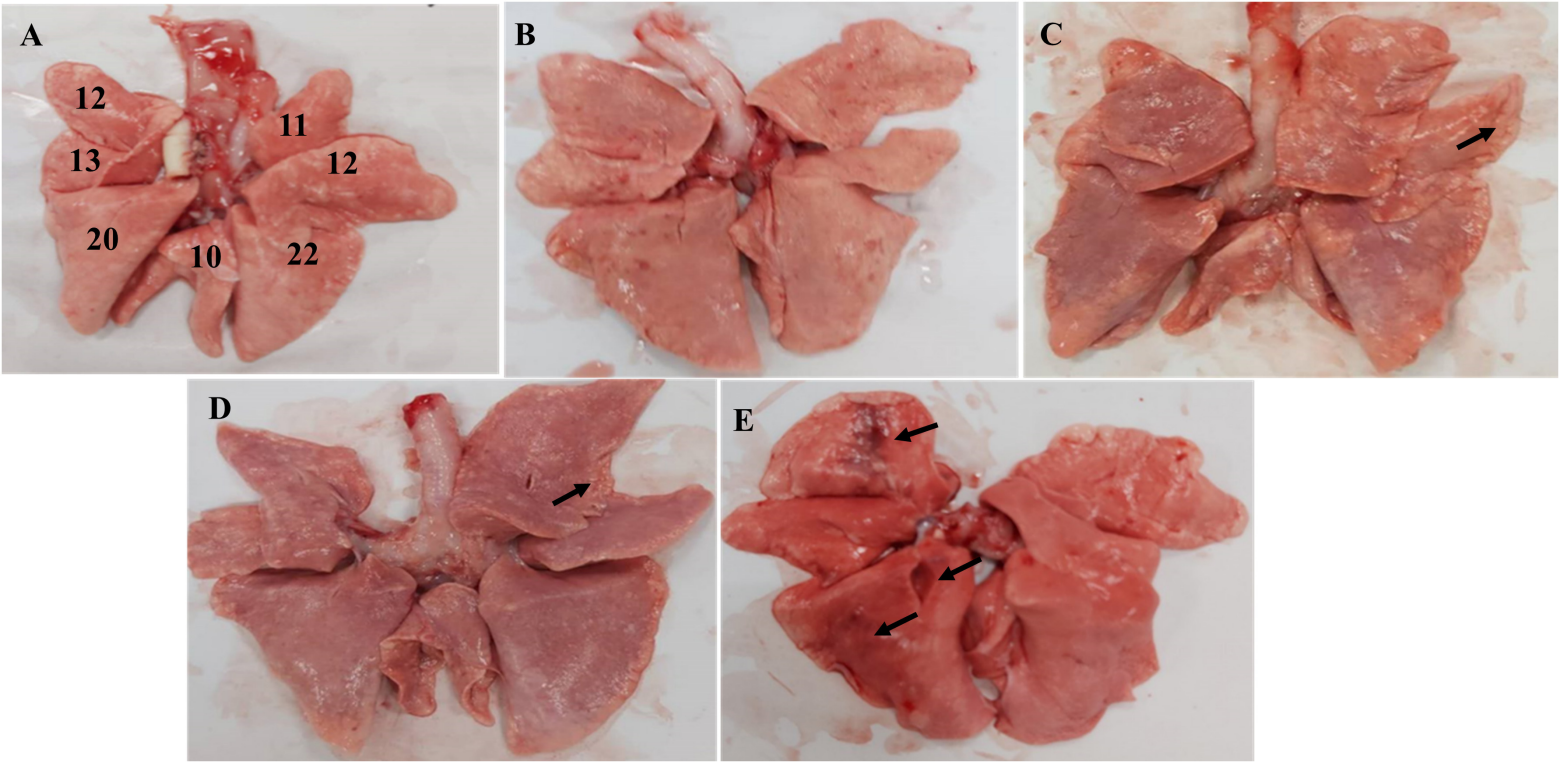
Macroscopic image of the Beagle lung. **(A)** Normal Beagle lung. Lungs of the **(B)** 10^3^ EID_50_; **(C)** 10^4^ EID_50_; **(D)** 10^5^ EID_50_; and **(E)** 10^6^ EID_50_ challenge groups. The arrows refer to tissue damage.

Anatomical examination of the lungs revealed that the 10^6^ EID_50_ group had obvious lesions, including those in the lung interlobular lobe and the septum intumescentia. The lung surfaces had visible bleeding spots (Figure 4E). However, visible lesions were hardly seen in the lungs of the other three challenge groups (Figures 4B–D). The area of the affected lung of the dogs increased as the challenge dose increased (Figure 2D). When the challenge dose was 10^6^ EID_50_, the area of the affected lung was >10%, indicating that this challenge dose, not 10^5^, 10^4^, or 10^3^ EID_50_, could successfully construct the H3N2 CIV infection model.

### Pathological Changes and IHC Analysis

**Figure 5:**
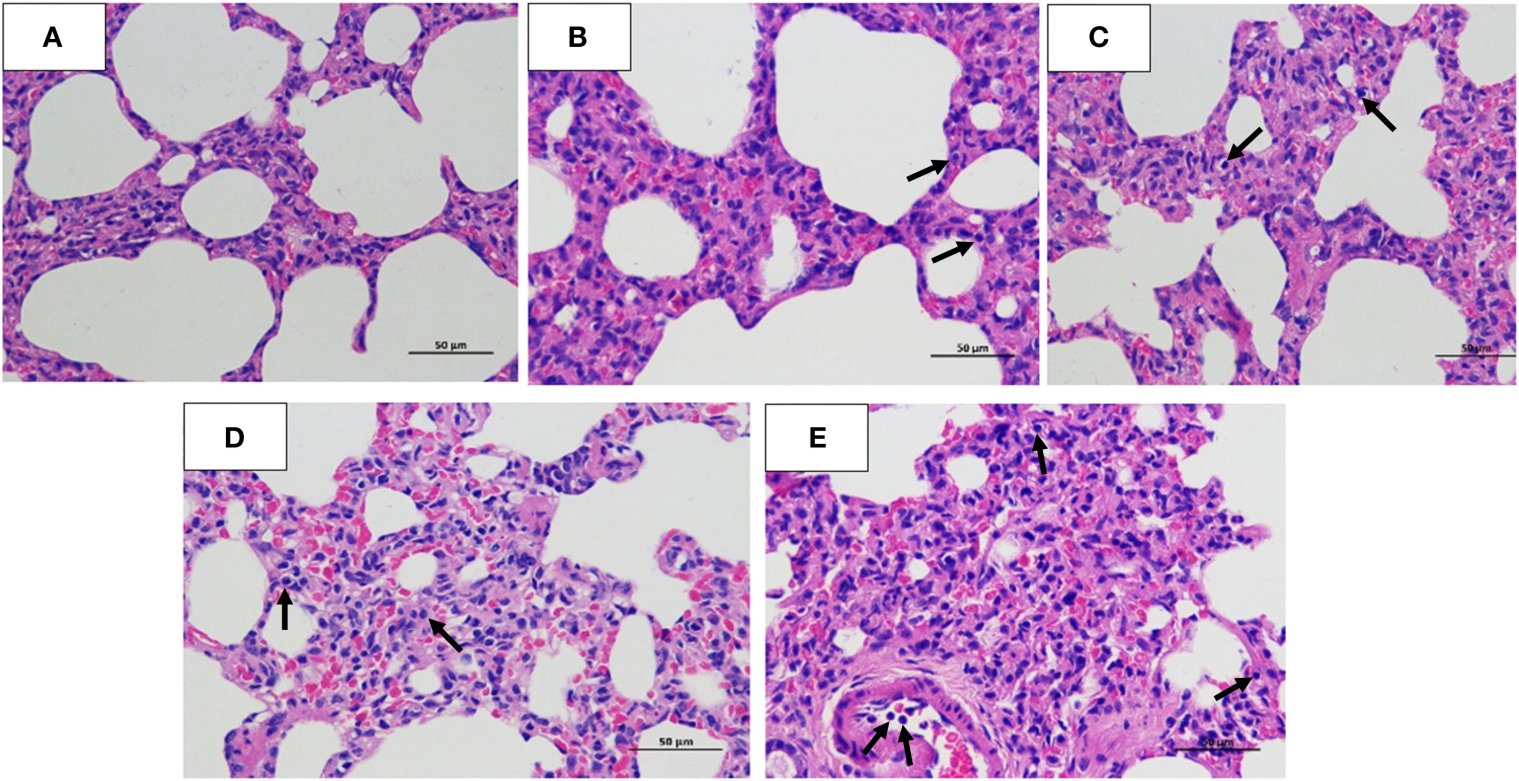
Hematoxylin and eosin staining. Lung tissues were collected for histopathology (hematoxylin and eosin) staining analysis at 5 dpi. **(A)** Normal Beagle lung histopathology. Pathological lung sections of the **(B)** 10^3^ EID_50_; **(C)** 10^4^ EID_50_; **(D)** 10^5^ EID_50_; and **(E)** 10^6^ EID_50_ challenge groups. The arrows refer to the infiltration of inflammatory cells.

**Figure 6:**
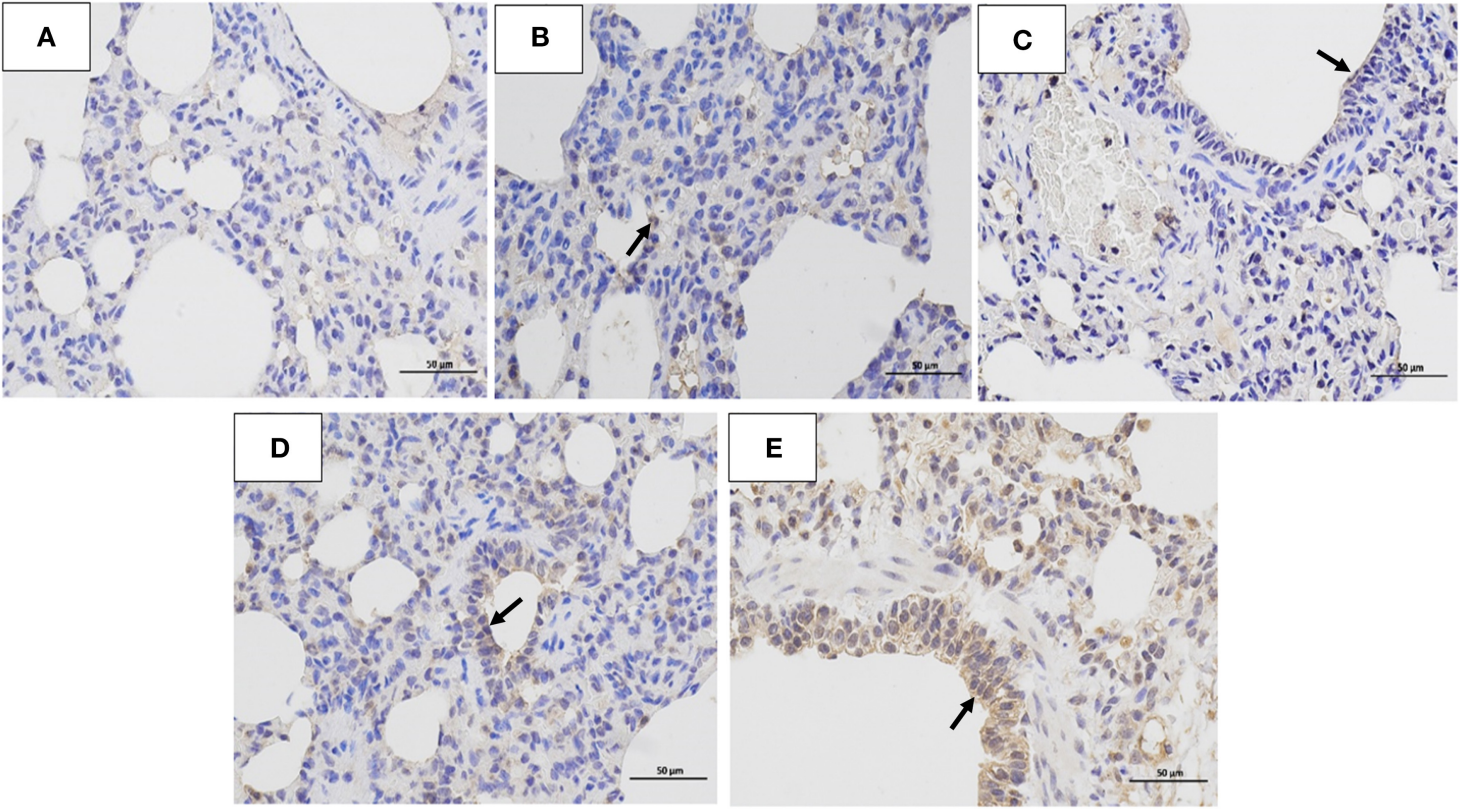
Immunohistochemistry analysis. Immunohistochemistry (IHC) analysis of the lungs of dogs challenged with different doses of H3N2 CIV: **(A)** control group; **(B)** 10^3^ EID_50_ challenge group; **(C)** 10^4^ EID_50_ challenge group; **(D)** 10^5^ EID_50_ challenge group; and **(E)** 10^6^ EID_50_ challenge group. The arrows refer to GD14 antigen expression.

The histopathology scores in the 10^6^ EID_50_ group were significantly higher than those in the 10^5^ EID_50_ group (*P* < 0.05, as shown in Figure 2E). Lung pathological lesions, including emphysema and pulmonary congestion, pulmonary interstitial hyperplasia, and narrow alveolar cavity, were observed in the 10^6^ EID_50_ group (Figure 5E) Tissue hyperemia and inflammatory reaction were more serious, with the infiltration of inflammatory cells such as granulocytes, plasma cells, and lymphocytes. When the challenge dose was 10^5^ EID_50_, only slight alveolar stenosis could be observed in the lungs of the dogs (Figure 5D). However, when the challenge dose was 10^4^ and 10^3^ EID_50_, there were no obvious pathological changes observed in the lungs (Figures 5B,C), with no differences compared with the control group. IHC analyses were conducted to determine the location of tissue damage and the distribution of the CIV antigen. As shown in Figure 6E, strong staining for CIV antigens was observed in the tracheal epithelial cells, bronchial epithelial cells, and alveolar epithelial cells of the 10^6^ EID_50_ group, while weak staining for CIV antigens was detected in the bronchial epithelial cells of the other three infection groups (Figures 6B–D).

## Discussion

Dogs can not only be infected by CIV but also by other IAV subtypes such as H3N8, H3N1, H1N1 (18). As a popular companion, dogs have close contact with humans and are therefore considered as potential intermediate hosts for IAVs (19). The main clinical symptoms of CIV include sneezing, coughing, running nose, tearing of the eyes, fever, conjunctivitis, and anorexia. In the case of CIV infection, histological examination often reveals bronchitis, bronchiolitis, and pneumonia (3, 20). H3N2 CIV is a new respiratory pathogen in dogs (21). Vaccination is the primary measure to prevent and control it (22).

Determining the minimum infectious dose of H3N2 CIV in Beagles is critical to the development of the CIV vaccine. In 2003, Richt et al. established a scoring standard for tissue damage after infection with the H3N2 swine influenza virus (17). Jirjis and Deshpande (13, 14) established the H3N8 CIV-challenged Beagles clinical symptoms and lung affected area scoring criteria. Based on our previous research (11, 23, 24), we developed the scoring criteria for clinical symptoms and lung tissue damage. To determine the minimum infectious dose of H3N2 CIV causing obvious clinical characterization and typical lung damage in Beagles, different doses of the virus were used to infect the Beagles.

The dose of 10^6^ EID_50_ inoculated intranasally caused obvious clinical symptoms and tissue damage compared to the other three doses. The 10^6^ EID_50_ group showed clinical symptoms at 2 dpi, which persisted longer than those in the other three groups. At the same time, the clinical symptom score was >3. To evaluate the immune response, HI antibody titers were tested after infection. At 9 dpi, antibodies were detected in all four experimental groups. This indicates that to resist virus invasion, the body induces an immune response against IAVs which often causes secondary bacterial diseases and lung diseases after infection (25). To assess the degree of lung injury caused by different challenge doses, we collected lung tissues from the dogs at 5 dpi. When the dose was 10^3^ EID_50_, 10^4^ EID_50_, and 10^5^ EID_50_, the lung injury was mild, not showing obvious symptoms. Conversely, when the dose was 10^6^ EID_50_, lung lesions were evident. We determined that the infectious dose of 10^6^ EID_50_ could cause moderate disease symptoms, with virus shedding for 7–8 days and tissue damage. Although the three groups infected with low doses of CIV did not show severe clinical signs, they could still shed the virus through the nasal cavity for 7 days. Therefore, these asymptomatic infected animals can still transmit CIV. Because we did not test for broad tissue tropism (except for the respiratory tract) and the presence of the virus in rectal swabs, we cannot tell whether CIV can be transmitted through the digestive tract, but it is reported that the virus can be detected in rectal swabs from dogs infected with H3N2 CIV (26), indicating the possibility of fecal-oral transmission of CIV.

Altogether our results show that 10^6^ EID_50_ is the minimum dose of H3N2 CIV to trigger obvious clinical manifestations in Beagles. When Beagles received an infectious dose of <10^6^ EID_50_, although the virus could be detected in the respiratory tract and nasal cavity, it usually did not cause noticeable clinical signs. Therefore, animal owners and veterinarians may not detect and treat a CIV infection in time, allowing the virus to circulate and further adapt in dogs. These results can provide valuable information for developing an H3N2 CIV infectious model in Beagles for subsequent vaccine development and prevention and treatment of CI.

## Data Availability Statement

All datasets generated for this study are included in the article/supplementary material.

## Ethics Statement

The animal study was reviewed and approved by Experimental Animal Ethics Committee of South China Agricultural University.

## Author Contributions

SL: conceptualization, funding acquisition, project administration, and visualization. CF: data curation and formal analysis. XW, SY, JO, LW, JL, GL, XZ, HX, JH, and PW: methodology. JL: software. XW, SY, JO, and JL: validation. YL: writing—original draft and writing—review and editing. All authors contributed to the article and approved the submitted version.

## Conflict of Interest

The authors declare that the research was conducted in the absence of any commercial or financial relationships that could be construed as a potential conflict of interest.
